# Water intake of pigs consuming tiamulin during the nursery phase^1^

**DOI:** 10.1093/tas/txab023

**Published:** 2021-02-08

**Authors:** Kimberly A Vonnahme, Adam Mueller, Daniel A Nelson, Manuel Alexander Vasquez-Hidalgo, Deborah Amodie, Thomas H Short, Martha A Mellencamp, Lucina Galina Pantoja

**Affiliations:** 1 Outcomes Research, Zoetis, Inc., 10 Sylvan Way, Parsippany, NJ 07054, USA; 2 Swine Services Unlimited, Inc. 6212 155th St NE, Rice, MN 56367, USA; 3 Pork Technical Services, Zoetis, Inc., 10 Sylvan Way, Parsippany, NJ 07054, USA

**Keywords:** nursery pigs, tiamulin hydrogen fumarate, water intake

## Abstract

Mass medication to manage population health can be achieved by providing therapeutics in the drinking water. Young nursery pigs are highly sensitive to the flavor and smell of water. Medications that reduce water palatability often lead to an interruption in water and feed intake. With the availability of several generic water-soluble antimicrobials for pigs, questions have arisen about their palatability compared with the original product. In this study, we compared the intake of water containing tiamulin hydrogen fumarate from two different manufacturers with the intake of unmedicated water. The hypothesis was that the intake of tiamulin-containing water would be similar to unmedicated water. Water intake was monitored upon entry into the nursery and just prior to leaving the nursery. Also, average daily gain (ADG) and feed efficiency (FE) were determined. A total of 300 pigs were individually weighed (4.2–10.9 kg; avg = 6.8 kg) for randomization to pen (*n* = 30 pens). The experiment had two time points: 1) early nursery (periods 1–3) and 2) late nursery (period 4). Pens were randomly assigned to a sequence (period 1–3) in a crossover experimental design containing three 10-d periods, with 5 d for the resetting of baseline where unmedicated water was provided followed by 5 d on tiamulin source addition [i.e., Triamulox^TM^ (Zoetis, Parsippany, NJ); Denagard (Elanco Animal Health, Greenfield, IN)] or unmedicated water. After period 3 was concluded, all pens were given unmedicated water (via nipple waterers) and the number of pigs per pen was reduced to six pigs to maintain adequate space per pig. Ten days prior to pigs leaving the nursery, a fourth period was performed. After a 5-d water baseline was achieved, pens were treated with either unmedicated water or Triamulox- or Denagard-containing water. Pigs had ad libitum access to water and feed. During the testing periods, daily water intake was measured by a cup water system in each pen. Feed intake was measured every 5 d. There was no effect of treatment on initial body weights or weights at the beginning or end of each period (*P* ≥ 0.51). Therefore, there was no effect of treatment on ADG (*P* ≥ 0.23). Water intake (*P* ≥ 0.16) and FE (*P* ≥ 0.35) were not affected by treatment. Water consumption was similar among all treatments in each of the four periods. There appears to be no aversion to water intake when tiamulin hydrogen fumarate is added to the drinking water.

## INTRODUCTION

Sufficient water intake is essential for pig health and productivity. Water intake can be influenced by physiological, biochemical, nutritional, and behavioral aspects but also by water quality. Water quality is determined by specific components within the water as well as the water delivery system within the livestock facility (Patience, 2013). The high dielectric constant of water gives it the ability to dissolve a wide variety of substances, including medications, and transport them through the body via the circulatory system (NRC, 2012). On many commercial pig farms, groups of growing pigs are mass medicated in the drinking water for short periods of time to manage herd health. However, proper administration of the medication is necessary to properly treat pigs and to avoid underdosing or overdosing, which may result in antibiotic resistance or toxicity, respectively (Little et al., 2019).

While there is data describing which water constituents have adverse impacts on water palatability and health (WHO, 2011), it is unclear what the effects of different pharmaceutical or additive products have on water acceptance and water intake. With the availability of several generic water-soluble antimicrobials for swine, questions have arisen regarding their palatability compared with the pioneer product counterpart. One water medication often used in nursery pigs for the treatment of swine pneumonia and dysentery is tiamulin hydrogen fumarate.

We hypothesized that the water intake of nursery pigs consuming water treated with either the generic or pioneer product containing tiamulin hydrogen fumarate would be similar and that the water intake of pigs consuming water treated with tiamulin would be similar to those consuming unmedicated water. Our objectives were to measure water and feed intake and the growth rate of pigs consuming tiamulin treated or unmedicated water in the nursery phase of production.

## MATERIAL AND METHODS

**Figure 1.**
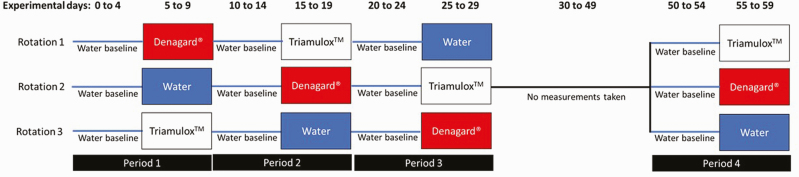
Experimental design for the study to investigate the impact of the addition of a tiamulin hydrogen fumarate product on water intake. The experiment had two time points: 1) early nursery (periods 1–3) and 2) late nursery (period 4). Pigs were weaned at 21 d (i.e., day 0 of the study) and randomly assigned to a sequence (periods 1–3) in a crossover experimental design containing three 10-d periods, with 5 d for the resetting of baseline where unmedicated water was provided followed by 5 d on tiamulin source addition [i.e. Triamulox (Zoetis, Parsippany, NJ); Denagard (Elanco Animal Health, Greenfield, IN)] or unmedicated water. Pens were also randomly assigned to their period 4 treatment prior to the initiation of the study. After period 3 was concluded, all pens were given unmedicated water (via nipple waterers) and the number of pigs per pen was reduced to six pigs to maintain adequate space per pig. Ten days prior to pigs leaving the nursery, a fourth period was performed. After a 5-d water baseline was achieved, pens were treated with either unmedicated water or Triamulox- or Denagard-containing water.

This study was approved by Swine Services Unlimited, Inc.’s (Rice, MN) Animal Care and Use Committee (SSUI19MAR18-5). High-health barrows (*n* = 300; 4.2–10.9 kg; avg = 6.8 kg) from a sole-source multiplier were weaned at 3 wk of age (day 0 of the study), and individually weighed for randomization to pen (2 barns with 15 pens/barn; *n* = 30 pens; 10 pigs/pen). The experiment had two time points: 1) early nursery (periods 1–3); and 2) 5 d prior to exit from the nursery (period 4). Pens were randomly assigned to a sequence (periods 1–3; Fig. 1) in a crossover experimental design. After a 5-d baseline measurement of water intake with pigs provided unmedicated water, there were three 10-d periods: 5 d on tiamulin source addition [i.e., Triamulox (Zoetis, Parsippany, NJ); Denagard (Elanco Animal Health, Greenfield, IN)] or unmedicated water, followed by 5 d for resetting of baseline (i.e., washout period) where unmedicated water was provided. Pens were also randomly assigned to their period 4 treatment prior to the initiation of the study. After period 3 was concluded, all pens were given unmedicated water (via nipple waterers) and the number of pigs per pen was reduced to six pigs to maintain adequate space per pig. Ten days prior to pigs leaving the nursery, a fourth period was performed. After a 5-d water baseline was achieved, pens were supplied unmedicated water or water containing either Triamulox or Denagard.

Both tiamulin sources were administered by their respective manufacturers’ specifications on the label for treating swine pneumonia [i.e., 681 mg (180 ppm) tiamulin hydrogen fumarate/3.79 L] for five consecutive days. Water (both unmedicated and treated) was weighed prior to delivery to its respective pens. Daily water disappearance was measured by a cup water system (15–19 L holding capacity per pen) within each pen. Every 24 h, water disappearance was recorded by draining and weighing the unconsumed water and subtracting that weight from the daily starting weight. Each waterer was observed at least once mid-day to ensure that enough water was present for ad libitum water throughout the day. Feed was also provided ad libitum.

At the beginning and end of each period, individual pig and feed weights were recorded to monitor growth and feed intake. All treatments consumed the same corn-soybean meal-based diets throughout the project.

### Calculations

As water consumption increased with body weight, water intake was expressed as a percentage of pen weight. Pen weight was obtained at the beginning of every 5-d period. When daily water consumption was calculated per body weight, the pen body weight obtained at the beginning of the 5-d period was used. Therefore, when daily water intake is expressed per body weight, the appearance that water intake changes over the 5 d is partially due to the pigs gaining weight over the 5-d period. For the total amount of water consumed over a 5-d period, daily water intake was summed and divided by pen weights at the beginning of the 5-d treatment period. Average daily gain (ADG) was calculated as gain divided by the total number of pig days. Feed efficiency (FE) was calculated as the weight gain of the pigs divided by the amount of feed consumed.

### Statistical Analyses

Periods 1–3 were analyzed together with sequence in the model. Since pens were rerandomized to treatments, period 4 was analyzed separately from the others. Water intake as a percentage of body weight was analyzed by a general linear mixed model approach for repeated measures. Using the SAS Proc Mixed Procedure (SAS 9.4, Cary NC), water intake as a percentage of body weight was analyzed with a model that considered the fixed effects of treatment, period, sequence, day, and the interactions of treatment-by-day, sequence-by-day, and period-by-day and the random effects of room, pen-within-sequence, and the residual error. Day was the repeated factor.

The covariance structure in the repeated measures analysis was investigated using six structural assumptions, namely, compound symmetry, heterogeneous compound symmetry, power, first-order autoregressive, heterogeneous first-order autoregressive, and unstructured. The assumption giving the minimum value of the Akaike’s information criterion was selected in the final analysis. Treatment least square means (LSMeans) were calculated for each group. Comparisons of LSMeans were performed by the two-sided Student’s *t*-test at the 5% level of significance.

Average daily gain and FE were analyzed by a general linear mixed model approach. Using the SAS Proc Mixed Procedure, these variables were analyzed with a model that considered the fixed effects of treatment, period, and sequence and the random effects of room, pen-within-sequence, and the residual error. Treatment LSMeans were calculated for each group and compared by the two-sided Student’s *t*-test at the 5% level of significance. For all variables of interest in periods 1–3, there was no effect of sequence rotation (*P* ≥ 0.59). All fixed effects were tested at the 5% level of significance.

## RESULTS

**Figure 2.**
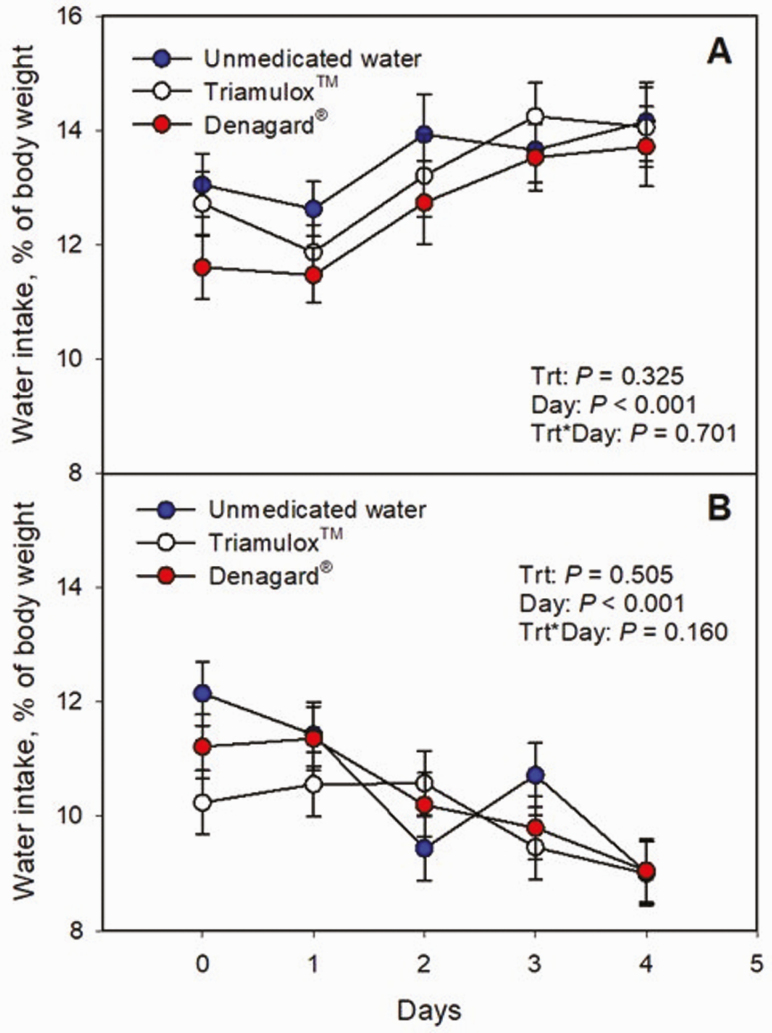
Daily water intake (as a percentage of total pen body weight) in periods 1–3 (A) and period 4 (B) in pigs provided unmedicated water (blue) or Triamulox (white) or Denagard (red) containing water. Body weights were obtained at the beginning of each 5-d testing period.

There was no effect (*P* ≥ 0.16) of water medication, or water medication source, on daily water intake over the 5-d treatment (Figs. 2A and 2B). The significance of the main effect of day was expected due to the denominator staying constant as pen weight was determined at the beginning of each period. Moreover, there was no effect (*P* ≥ 0.30) of treatment on total water consumed as a percentage of pen weight (Tables 1 and 2). Lastly, there was no effect (*P* ≥ 0.23) of treatment on ADG or FE during the duration of the project due to the addition of a tiamulin-containing product in the water (Tables 1 and 2).

**Table 1.** Effect of tiamulin hydrogen fumarate products added to water on water intake, ADG, and FE in the first 30 d in the nursery (i.e. periods 1–3)

	Unmedicated	Triamulox	Denagard	SEM	*P* value
Initial pig weight, kg	13.13	12.80	12.94	0.43	0.512
Water consumed, % of body weight	13.49	13.22	12.62	0.42	0.309
ADG, kg/d	0.48	0.54	0.53	0.03	0.301
Gain:feed	0.63	0.71	0.70	0.04	0.355

LSMeans ± SEM are presented. Periods 1–3 were conducted from day 0 (i.e., weaning, 21 d of age) to day 29. There were 10 pens per treatment, each treatment was replicated thrice (i.e., *n* = 30 per treatment). There were 10 barrows per pen. There was no period × treatment interaction. Pigs were heavier (*P* < 0.01) with each subsequent period. After a 5-d baseline measurement of water intake with pigs provided unmedicated water, there were three 10-d periods: 5 d on tiamulin source addition (i.e., Triamulox, Denagard, or unmedicated water), followed by 5 d for resetting of baseline (i.e., washout period) where unmedicated water was provided.

**Table 2.** Effect of tiamulin hydrogen fumarate products added to water on water intake, ADG, and FE in the last 10 d in the nursery (i.e., period 4)

	Unmedicated	Triamulox	Denagard	SEM	*P* value
Initial pig weight, kg	44.06	44.03	44.28	1.07	0.984
Water consumed, % of body weight	10.55	9.97	10.32	0.35	0.505
ADG, kg/d	0.81	0.92	0.91	0.05	0.231
Gain:feed	0.49	0.54	0.51	0.03	0.427

LSMeans ± SEM are presented. Period 4 was conducted from days 50 to 59 (day 0 = weaning). There were 10 pens per treatment. There were six barrows per pen. After a 5-d baseline measurement of water intake with pigs provided unmedicated water, pens had 5 d on tiamulin source addition (i.e., Triamulox, Denagard, or unmedicated water).

## DISCUSSION

We accept our hypothesis that the inclusion of a tiamulin medication in drinking water does not impact water consumption by nursery pigs. Moreover, the addition either in the generic or original tiamulin-containing product does not impact water palatability as daily water intake was not affected by the addition of either product.

In growing pigs, water intake has been estimated to be 80–120 mL of water per kilogram of body weight (NRC, 2012). Our data agree with this range of water consumption. Water intake increases as feed intake increases; however, the feed to water ratio decreases (Barber et al., 1991). Our study confirms this finding as there was ~0.40 kg of feed consumed per pig per day in periods 1–3 and, by period 4, it increased to ~0.80 kg of feed consumed per pig per day, with water intake decreasing by ~12% during this same duration. Pigs are prandial drinkers with 75–85% of their drinking events being related with meals (Little et al., 2019). Therefore, the best predictors for water intake are dry feed intake (NRC 2012) and metabolic body weight (Brooks et al., 1984). Water consumption is driven by satiety and influenced by factors such as stress, boredom, hunger, environmental temperature, and feed type (Little et al., 2019). Therefore, predicted water intake should always include diet composition and environmental influences (Brooks et al., 1984). It should be noted that the addition of tiamulin hydrogen fumarate did not alter water or feed consumption in the current controlled study.

The effect of antibiotic addition to water versus feed has been studied in the past in order to analyze if water-administered antibiotics would be as effective as feed-added antibiotics (Brooks et al., 1984). Some illnesses depress feed intake and can reduce medication intake but, in respiratory diseases, water intake is not adversely affected (Brooks et al., 1984). Similarly, feed intake was decreased in weaned pigs consuming feed medicated with carbadox (Maenz et al., 1993). In rats, the inclusion of enrofloxacin, amoxicillin, sulfa-trimetoprim, and doxycycline in water did not influence water consumption (Marx et al., 2014). To our knowledge, there is limited information in pigs about the effects of water-added antibiotics on water intake.

While there is little known how water medications impact water intake, there are more publications on the effect of antibiotics in feed. Brooks and Carpenter (1990) reported that the type of antibiotic, and the circumstances in which it is fed, may produce differing results. They reported that water intake varies depending upon the antibiotic (i.e., penicillin and aureomycin) that is added to the feed (Brooks and Carpenter, 1990). This is similar to another study in which the addition of furazolidone and tylosine to the feed did not affect water intake in growing pigs (McLeese et al., 1992). On the other hand, Brooks and Carpenter (1990) also reported that the addition of oxytetracycline to diets increased water demand by 13.7%. It has been hypothesized that the impact of antibiotics on water intake will depend upon the relative extent to which water loss is reduced by the control of gastrointestinal disruption (i.e., diarrhea) and that water intake may be increased to enable renal clearance of the antibiotic or its residues (Brooks and Carpenter, 1990).

There are certain antibiotics that have a greater solubility, thus, availability, in water than others (Little et al., 2019). Solubility is affected by chemicals and minerals in the water, water temperature, and pH (Little et al., 2019). It seems that the most important aspect of antibiotics in water is the amount of antibiotic that is consumed and how much of that antibiotic is bioavailable. Factors such as the type of antibiotic and the amount of feed present in the gastrointestinal system can influence antibiotic absorption and are key in the efficacy of water-added antibiotics (Little et al., 2019). The most important question to ask when adding antibiotics to the drinking water appears to be how much of the antibiotic ultimately makes it into the plasma and if these plasmatic concentrations are therapeutic.

It has been suggested that the physiological and ethological needs of pigs can only be satisfied by the provision of an unrestricted supply of water at all times (Thulin and Brumm, 1991). When mass treating pigs for respiratory diseases as they enter the nursery, it is important that the supply of freshwater is adequate at all times. Inadequate access to water can negatively affect ADG (Thulin and Brumm, 1991). Conditions in which water deprivation happens (i.e., frozen water pipes, faulty equipment, and low water pressure) can cause gastrointestinal disturbances, such as diarrhea that ultimately end in death (Brooks and Carpenter, 1990).

The proper dosage of a tiamulin hydrogen fumarate-containing product does not influence water palatability, or the water intake of growing pigs, when administered either upon entry or just prior to exit of the nursery. A readily available source of good-quality water should supply the required water requirements of the growing pig, and when the need for short mass medications occurs, water intake will not be altered by a tiamulin source.
